# Comparison of the Biological Characteristics of Mesenchymal Stem Cells Derived from Bone Marrow and Skin

**DOI:** 10.1155/2016/3658798

**Published:** 2016-04-27

**Authors:** Ruifeng Liu, Wenjuan Chang, Hong Wei, Kaiming Zhang

**Affiliations:** ^1^Institute of Dermatology, Taiyuan City Centre Hospital, Shanxi Provincial Key Laboratory of Immunological Skin Diseases, No. 1 Dong San Dao Xiang, Taiyuan, Shanxi 030009, China; ^2^Department of Dermatology, Zibo City First Hospital, Shandong 255200, China

## Abstract

Mesenchymal stem cells (MSCs) exhibit high proliferation and self-renewal capabilities and are critical for tissue repair and regeneration during ontogenesis. They also play a role in immunomodulation. MSCs can be isolated from a variety of tissues and have many potential applications in the clinical setting. However, MSCs of different origins may possess different biological characteristics. In this study, we performed a comprehensive comparison of MSCs isolated from bone marrow and skin (BMMSCs and SMSCs, resp.), including analysis of the skin sampling area, separation method, culture conditions, primary and passage culture times, cell surface markers, multipotency, cytokine secretion, gene expression, and fibroblast-like features. The results showed that the MSCs from both sources had similar cell morphologies, surface markers, and differentiation capacities. However, the two cell types exhibited major differences in growth characteristics; the primary culture time of BMMSCs was significantly shorter than that of SMSCs, whereas the growth rate of BMMSCs was lower than that of SMSCs after passaging. Moreover, differences in gene expression and cytokine secretion profiles were observed. For example, secretion of proliferative cytokines was significantly higher for SMSCs than for BMMSCs. Our findings provide insights into the different biological functions of both cell types.

## 1. Introduction

Mesenchymal stem cells (MSCs) are adherent stromal cells that were first isolated from the bone marrow [1] and are characterized by their ability to differentiate into mesenchymal tissues such as bone, cartilage, and fat. In addition, MSCs have been shown to suppress immune responses [2–5]. Because of these properties, MSCs have recently gained increasing attention from researchers and have now been shown to be present in a variety of tissues, including the umbilical cord, placenta, adipose tissues, and skin [6–11]. MSCs derived from different tissues may have some unique biological characteristics.

In a previous study, we found that the biological behaviors of bone marrow MSCs (BMMSCs) in patients with psoriasis were abnormal [12, 13]. Because psoriasis is a type of skin disease associated with immune abnormalities, the biological characteristics of MSCs from psoriatic skin lesions may more accurately reflect the features of psoriasis. Indeed, analysis of MSCs from psoriatic lesions showed that these cells exhibit abnormalities in gene expression, cytokine secretion, and immune properties [14–16]. Moreover, BMMSCs and MSCs isolated from skin (SMSCs) have been shown to have different properties. Although the methods for isolation and culture of BMMSCs have been extensively studied, culture methods for SMSCs are not yet optimized, and some researchers believe that SMSCs may actually be fibroblasts [17].

Therefore, in the current study, we performed a comprehensive comparison of MSCs from the two sources, including analysis of the skin sampling area, separation method, culture conditions, primary and passage culture times, cell surface markers, multipotency, cytokine secretion, gene expression, and fibroblast-like features.

## 2. Material and Methods

### 2.1. Participants

All volunteers provided informed consent for their participation in the study. The protocol involving human subjects was approved by the Medical Ethics Committee of Taiyuan City Centre Hospital and was performed in accordance with the 1964 Declaration of Helsinki and its later amendments or comparable ethical standards.

Twenty bone marrow samples were from normal bone marrow donors, and 20 sex- and age-matched volunteers from the Urology and Plastic Surgery Department, Taiyuan City Centre Hospital, were enrolled in this study.

### 2.2. Reagents

Cell culture plates and plastic flasks were purchased from Corning Incorporated (Corning, NY, USA). Dulbecco's modified Eagle's medium (DMEM)/F12 medium, B-27 supplement, fetal bovine serum (FBS), and Percoll were purchased from Invitrogen (Grand Island, NY, USA). Trypsin, dispase enzyme II, recombinant human basic fibroblast growth factor (bFGF), and toluidine blue were purchased from Sigma-Aldrich (St. Louis, MO, USA). Mouse monoclonal antibodies against human stem cell factor (SCF), granulocyte colony-stimulating factor (G-CSF), granulocyte-macrophage colony-stimulating factor (GM-CSF), macrophage colony-stimulating factor (M-CSF), interleukin-1 (IL-1), IL-3, IL-6, IL-7, IL-8, IL-11, epidermal growth factor (EGF), vascular endothelial growth factor (VEGF), tumor necrosis factor-*α* (TNF-*α*), leukemia inhibitory factor (LIF), hepatocyte growth factor (HGF), and transforming growth factor-*β*1 (TGF-*β*1), as well as horseradish peroxidase- (HRP-) labeled rabbit antibodies against mouse IgG, were obtained from Abcam (Cambridge, UK). Phycoerythrin- (PE-) or fluorescein isothiocyanate- (FITC-) labeled mouse antibodies against human CD29, CD44, CD73, CD90, CD105, CD14, CD34, CD45, and human leukocyte antigen- (HLA-) DR were purchased from BD Biosciences (San Jose, CA, USA). The IMT2 inverted phase-contrast microscope was obtained from Olympus (Tokyo, Japan). Type 352 automatic microplate reader was obtained from Labsystems (Helsinki, Finland), and the EPICS-XL Flow Cytometer FACSCalibur was obtained from Beckman Coulter (Los Angeles, CA, USA).

### 2.3. MSC Separation and Cultivation and Measurement of the Culture Time

Human BMMSCs were grown from aspirates taken from the posterior superior iliac spine of healthy volunteers. Five milliliters of heparinized aspirate was diluted 1 : 2 with DMEM/F12 medium and centrifuged through a Percoll density gradient at 700 ×g for 20 min. The mononuclear cells at the interface were collected, washed twice with DMEM/F12 medium, resuspended at a concentration of 1 × 10^6^ cells/mL in complete medium (DMEM/F12 supplemented with 10% FBS, 100 U/mL penicillin, and 100 *μ*g/mL streptomycin), and plated at 1 × 10^6^ cells/well in 24-well plates. The cells were incubated at 37°C in a humidified atmosphere supplemented with 5% CO_2_. Two days later, nonadherent cells were removed by replacing the medium. Half of the medium was then changed every 4 days. At 90% confluency, the cells were detached by incubation with 0.25% trypsin, diluted 1 : 2 with complete medium, and then at 5 × 10^4^ cells/well in 24-well plates. Cell growth was observed daily under an inverted phase-contrast microscope; growth morphology, the level of confluence, and the culture time were recorded. The culture time for primary cells was defined as the time from inoculation of mononuclear cells to 90% confluence, the culture time for cells of passage 1 was defined as the time from inoculation of passage 1 cells to 90% confluence, and so forth.

For isolation of SMSCs, skin specimens were cut into 1 mm^3^ tissue blocks under sterile conditions and then digested with 0.25% dispase enzyme II at 37°C for 2–4 h. The epidermis and dermis were separated mechanically; the dermis was collected and finely minced. DMEM/F12 medium containing 10% FBS was added to the minced dermis, and the cells were separated by pipetting. After filtering through a 40 *μ*m aperture sieve, the filtrate was allowed to stand on ice for 20–30 min, after which it was centrifuged at 200 ×g for 5 min. The supernatant was discarded and the filtrate was added to culture medium to resuspend the cells. The cells were then cultured in DMEM/F12 medium supplemented with 10% FBS, 10 ng/mL bFGF, 20 *μ*L/mL B27 supplement, 100 U/mL penicillin, and 100 *μ*g/mL streptomycin. The culture was inoculated into T25 plastic flasks at a density of 1 × 10^5^ cells/cm^2^ and kept in an incubator at 37°C, with 5% CO_2_ and saturated humidity. After 72 h, the medium was removed, and all suspended cells were discarded. Freshly prepared medium, as described above, was added to continue the cultivation of the adherent cells. Half of the medium was replaced every fifth day. When cells had grown to nearly 90% confluence, they were digested with 0.25% trypsin and transferred to subcultures at 5 × 10^4^ cells/well in 24-well plates. After passaging, the cells were cultured in the medium as described above but without bFGF. Cell growth was observed daily under an inverted phase-contrast microscope; growth morphology, the level of confluence, and the culture time were recorded. The culture time for primary cells was defined as the time from cell inoculation to 90% confluence, the culture time for cells of passage 1 was defined as the time from inoculation of passage 1 cells to 90% confluence, and so forth.

### 2.4. Cultivation of Skin Fibroblasts

To differentiate between SMSCs and skin fibroblasts and to ascertain whether the cultivated cells were MSCs rather than fibroblasts, we cultured skin fibroblasts using explant culture techniques [18]. Skin specimens were cut into 2 mm^3^ blocks and washed twice with phosphate-buffered saline (PBS) containing antibiotics. After seeding the specimen blocks into T25 flasks using an aspirator, they were distributed evenly on the bottom of the flasks. The optimal distance between each seeded block was determined to be 0.5 cm, and each flask contained 20 specimen blocks. The flasks were tilted and filled with 0.5 mL of DMEM/F12 containing 10% FBS and then kept in an incubator at 37°C with 5% CO_2_ and saturated humidity for 4 h. After the tissues adhered to the surface, an additional 4 mL of DMEM/F12 containing 10% FBS was added carefully to prevent resuspension of the tissue blocks. The flasks were returned to the incubator, and the culture medium was changed every 3-4 days. After the seeded cells had grown to 90% confluence, they were passaged and subcultured into 24-well culture plates.

### 2.5. Identification of Cell Purity and Collection of Cell Medium

BMMSCs at passage 3 or SMSCs at passage 5 and their culture supernatants were collected from each of the 20 wells. Culture supernatants were stored in sterile tubes at −20°C after filtering through a 0.45 *μ*m filter for enzyme-linked immunosorbent assays (ELISAs) to determine the cytokine contents. Cells (BMMSCs at passage 3, SMSCs and skin fibroblasts at passage 5) were detached with 0.25% trypsin, washed, and resuspended in PBS. Cells (2 × 10^5^) were incubated in the dark with PE- or FITC-labeled mouse antibodies against the human cell surface markers CD29, CD44, CD73, CD90, CD105, CD14, CD34, CD45, and HLA-DR for 30 min. After washing with PBS, the cells were subjected to two-color flow-cytometric analysis to examine the proportion of cells positive for the respective antigens.

### 2.6. Multipotent Differentiation of MSCs and Identification of the Differentiated Cells

BMMSCs at passage 3 or SMSCs at passage 5 were induced to differentiate into lipocytes, osteoblasts, or chondrocytes. The specific induction methods were described previously [15]. After adipogenic differentiation for 10 days, the cells were fixed with 10% formalin, washed with 60% isopropyl alcohol, and stained with oil red O. After osteogenic differentiation for 3 weeks, the cells were fixed with 10% formalin and stained with 2% alizarin red solution. After chondrogenic differentiation for 21 days, micromasses were fixed with 4% paraformaldehyde, embedded in optimal cutting temperature compound, cut into 5 *μ*m sections, and stained with toluidine blue. Negative controls, for which differentiation-inducing supplements were omitted from the culture medium, were included for each differentiation assay.

The fifth-passage fibroblasts were induced to differentiate into fat, bone, and cartilage as described above.

### 2.7. Quantification of Cytokines Secreted into the MSC Medium

Cytokine content was measured based on direct ELISA. A 96-well plate was coated overnight at 4°C with 50 *μ*L medium from BMMSC and SMSC cultures per well. After washing of the plate, 200 *μ*L of 0.25% gelatin was added per well, and the plate was incubated for 2 h at room temperature (RT). Primary antibodies (50 *μ*L, diluted 1 : 100) were introduced into the wells, and the plates were incubated for 1 h at RT. After washing away excess primary antibody, 50 *μ*L of HRP-labeled secondary antibody (diluted 1 : 1000) was added to the wells and incubated for 45 min at 37°C. Finally, after washing off excess labeled antibody, HRP enzyme activity was determined by the o-phenylenediamine dihydrochloride reaction, which was terminated by adding 1 M H_2_SO_4_ after incubation for 10 min at RT. The concentration of each cytokine was calculated using CurveExpert Basic 1.40 software (https://www.curveexpert.net/).

### 2.8. Microarray Analysis

Total RNA was purified from each sample (*n* = 8) using an RNeasy mini kit (Qiagen Valencia, CA, USA) according to the manufacturer's instructions. RNA integrity was assessed using standard denaturing agarose gel electrophoresis. RNA quantity and quality were evaluated using NanoDrop ND-1000 spectrophotometer (NanoDrop Technologies, Wilmington, DE, USA).

The RNA was amplified and labeled using an Agilent Low Input Quick Amp Labeling Kit (Agilent Technologies, Waldbronn, Germany) and then hybridized to the Agilent Whole Human Genome Oligo Microarray. The array data were extracted using the Agilent Feature Extraction software (version 10.7.3.1). Global mean normalization was performed, and the probes with a signal intensity <800 or coefficient of variation of intensity of <20% in all samples, which represent the low-abundance and housekeeping genes, respectively, were selected for further analysis. Unsupervised hierarchical cluster analysis was performed using Cluster 3.0 software (http://www.falw.vu/~huik/cluster.htm). Differentially expressed genes (fold change, >2.0) with statistical significance (*p* < 0.05) were identified using volcano plot filtering. Significant enrichment of gene ontology (GO) terms was analyzed using the hypergeometric distribution in the R language package software (https://cran.r-project.org/), with statistical thresholds of *p* < 0.05 and false discovery rate (FDR) < 0.05.

### 2.9. Statistical Analysis

Data were expressed as the mean ± SD. Independent sample *t*-tests were used to compare the mean values of samples from bone marrow and skin in SPSS16.0 software (SPSS Inc., Chicago, IL, USA). Differences with *p* values of less than 0.05 were considered statistically significant.

## 3. Results

### 3.1. Morphological Features and Culture Times of BMMSCs and SMSCs

**Figure 1:**
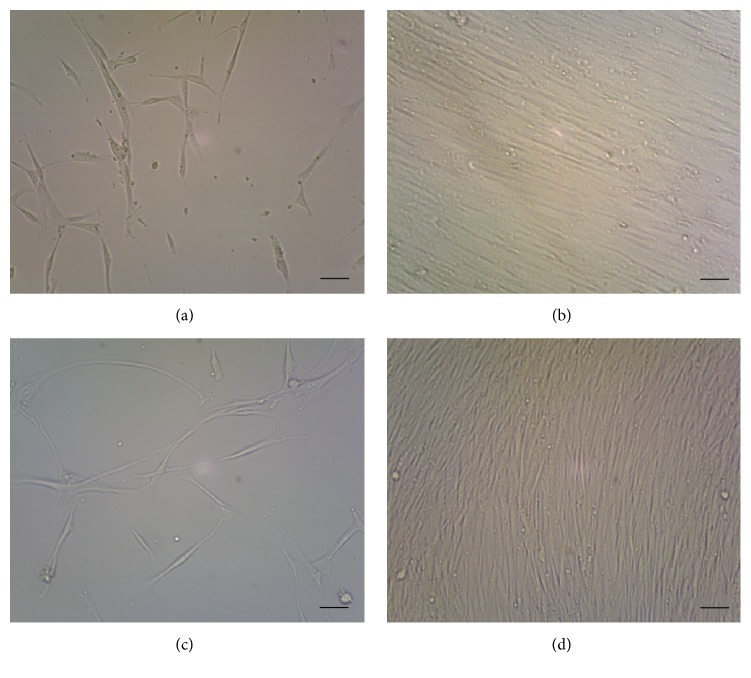
Morphological characteristics of bone marrow mesenchymal stem cells (BMMSCs) and skin mesenchymal stem cells (SMSCs). (a) BMMSCs cultured for 7 days. (b) BMMSCs cultured for 16 days. (c) SMSCs cultured for 16 days. (d) SMSCs cultured for 29 days. Scale bar: 10 *μ*m.

The cell morphologies of BMMSCs and SMSCs were similar. Isolated BMMSCs attached to the bottoms of the plates after incubation for 24 h. On days 7–10, the cells showed obvious enlargement and proliferation, forming small colonies with several to tens of fusocellular, triangular, and polygonal cells. The cells displayed typical fibroblast morphology with multilayered flat cell bodies having short cell processes connected to adjacent cells (Figure 1(a)). At approximately day 16, the cells reached 90% confluence (Figure 1(b)). When treated with trypsin, they became round; after reattachment to the plate and incubation for 24 h, the cell morphology reverted to the primary BMMSC shape. The cells reached 90% confluence after incubation for an average of 12 days.

A small number of adherent cells appeared 72 h after primary SMSCs were seeded; these cells then gradually increased in number and became significantly larger. A few cells were triangular or polygonal in shape; however, most were basically short or long spindle-shaped and had a fibroblast-like morphology. Cell bodies were enlarged and had cytoplasmic projections of various lengths and sizes, which were interconnected; the cells overlapped and proliferated in a stratified fashion, as shown in Figure 1(c). The time required for cultivation of the primary cell culture to 90% confluence was 29 days (Figure 1(d)). Subcultured cells had rounded shapes after digestion with 0.25% trypsin but returned to their original shapes after 24 h, showing adherence and proliferation, while retaining morphologies similar to those of the primary cells. The required level of confluence was reached within 3-4 days.

Table 1 shows the culture times of BMMSCs and SMSCs. The primary culture time of BMMSCs was significantly shorter than that of SMSCs (16.35 ± 4.38 versus 28.85 ± 5.52 days, resp.; *p* < 0.001). However, the growth rate of BMMSCs was lower than that of SMSCs after passage (time of passage 1, 12.25 ± 4.49 versus 3.85 ± 1.09 days; *p* < 0.001).

### 3.2. Identification of BMMSCs, SMSCs, and Fibroblasts

**Figure 2:**
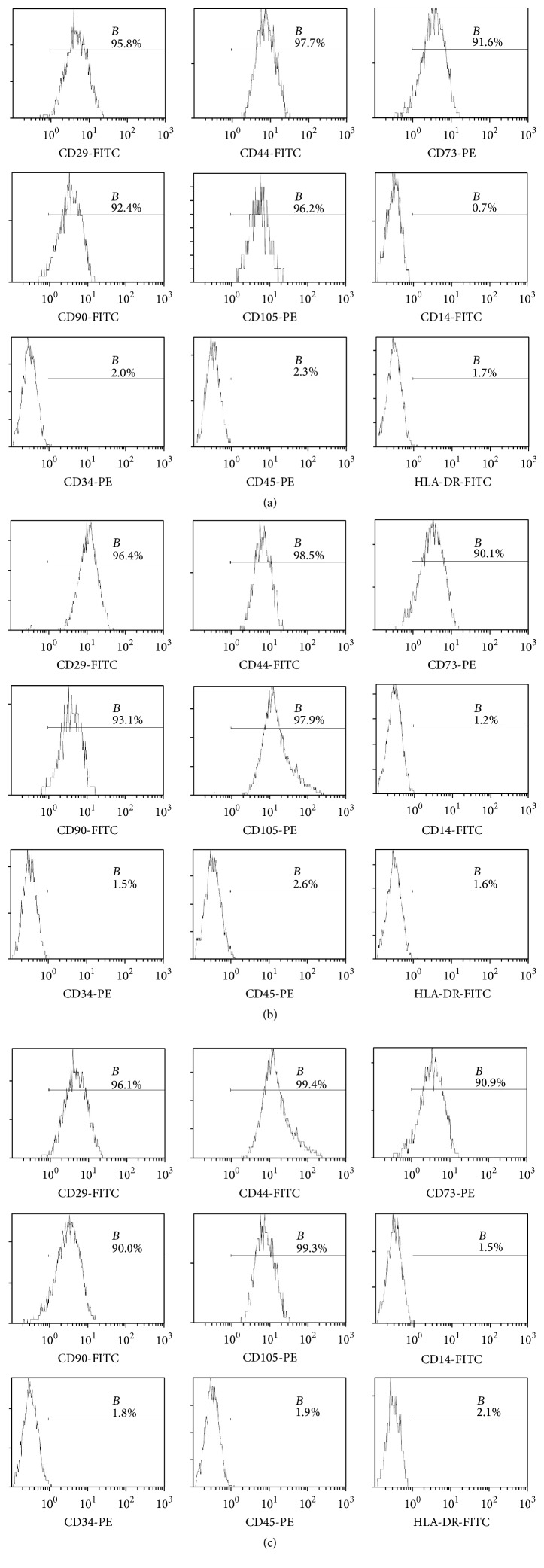
Phenotype identification of bone marrow mesenchymal stem cells (BMMSCs), skin mesenchymal stem cells (SMSCs), and skin fibroblasts. (a) Surface markers on BMMSCs. (b) Surface markers on SMSCs. (c) Surface markers on skin fibroblasts. The cells were positive for CD29, CD44, CD73, CD90, and CD105 and negative for CD14, CD34, CD45, and HLA-DR.

**Figure 3:**
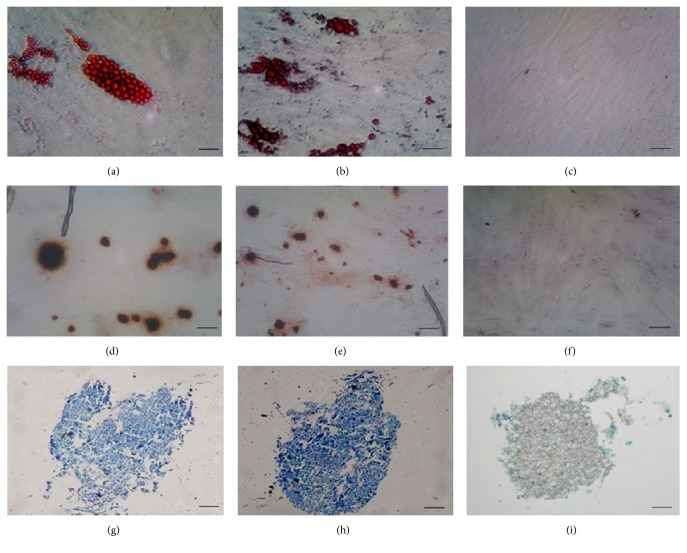
Morphological characteristics of lipocytes, osteoblasts, and chondrocytes induced to differentiate from bone marrow mesenchymal stem cells (BMMSCs), skin mesenchymal stem cells (SMSCs), and skin fibroblasts. (a) Lipocytes induced to differentiate from BMMSCs. (b) Lipocytes induced to differentiate from SMSCs. (c) Skin fibroblasts maintained the original cell morphology and were not stained by oil red O after induction to lipocytes. (d) Osteoblasts induced to differentiate from BMMSCs. (e) Osteoblasts induced to differentiate from SMSCs. (f) Skin fibroblasts maintained the original cell morphology and were not stained by alizarin red solution after induction to osteoblasts. (g) Chondrocyte pellet induced to differentiate from BMMSCs. (h) Chondrocyte pellet induced to differentiate from SMSCs. (i) Skin fibroblast micromasses were not stained by toluidine blue after induction to chondrocytes. Scale bar: 50 *μ*m.

Flow cytometry results showed that the purity of BMMSCs at passage 3 reached up to 90%, whereas that of SMSCs reached only 70%. The purity of SMSCs was more than 90% after the fifth passage. Expansion of BMMSCs results in gradual loss of osteogenic potential after passages 5-6 [19]. Therefore, in order to guarantee the purity of the cells and avoid the loss of cell biological characteristics as a result of passaging, we used BMMSCs at passage 3 and SMSCs at passage 5 for follow-up experiments. Flow-cytometric analysis of surface antigens of both groups of MSCs and fibroblasts showed high expression levels of CD29, CD44, CD73, CD90, and CD105 and negative expression of CD14, CD34, CD45, and HLA-DR in all three cell types (Figure 2). The MSCs all differentiated into the relevant cells and tissues after adipogenic, osteogenic, and chondrogenic induction (Figures 3(a), 3(b), 3(d), 3(e), 3(g), and 3(h)), indicating that the isolated and cultured cells met the identification criteria for MSCs [20]. Control cells did not show these important stem cell characteristics (data not presented). However, the skin fibroblasts of passage 5 maintained the original cell morphology after induction, and no positive results were observed after staining (Figures 3(c), 3(f), and 3(i)). These data suggested that fibroblasts were not differentiated into fat, bone, and cartilage.

### 3.3. Differential Secretion of Cytokines from BMMSCs and SMSCs

**Figure 4:**
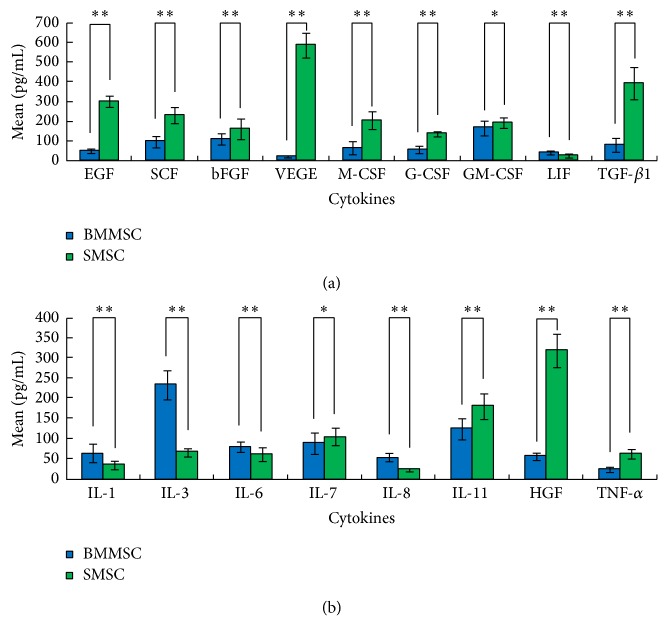
Cytokine levels in the culture medium of bone marrow mesenchymal stem cells (BMMSCs) and skin mesenchymal stem cells (SMSCs). (a) Cell proliferative cytokines. (b) Inflammatory cytokines. Cytokines from BMMSCs are indicated in blue; cytokines from SMSCs are indicated in green. ^*∗*^ *p* < 0.05; ^*∗∗*^ *p* < 0.01.

Table 2 shows the cytokine contents in culture media from BMMSC and SMSC cultures, as measured by direct ELISA. Among cell proliferative cytokines, the concentrations of EGF, SCF, bFGF, VEGF, M-CSF, G-CSF, GM-CSF, and TGF-*β*1 secreted from BMMSCs were significantly lower than those from SMSCs. In contrast, only LIF secretion from BMMSCs was significantly higher than that from SMSCs (Figure 4(a)). Among the inflammatory cytokines, the concentrations of IL-1, IL-3, IL-6, and IL-8 secreted from BMMSCs were significantly higher than those secreted from SMSCs. In contrast, the concentrations of IL-7, IL-11, HGF, and TNF-*α* secreted from BMMSCs were significantly lower than those secreted from SMSCs (Figure 4(b)).

### 3.4. Microarray Analysis

**Figure 5:**
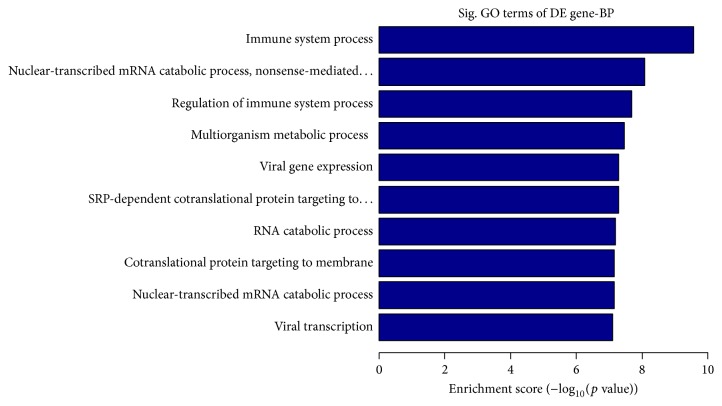
Enrichment scores of genes differentially expressed between bone marrow and skin mesenchymal stem cells determined by gene ontology analysis.

To identify the molecular phenotypes of BMMSCs and SMSCs, we used microarray analyses to identify differentially expressed genes (DEGs). A total of 810 genes were differentially expressed between BMMSCs and SMSCs. Among them, 652 genes showed higher and 158 showed lower expression in BMMSCs than in SMSCs. These DEGs were mainly involved in “immune system process,” “nuclear-transcribed mRNA catabolic process, nonsense-mediated decay,” and “regulation of immune system process” among other functions (Figure 5). Of the 141 DEGs involved in the immune system process, 82 DEGs are also involved in the regulation of this process. The majority (71 genes) of these 82 genes were highly expressed in BMMSCs (Table 3).

## 4. Discussion

MSCs are important stem cells that exhibit high proliferation and self-renewal capabilities. Because MSCs can differentiate into various cell types, they are important for tissue repair and regeneration during ontogenesis [21, 22]. Owing to their high proliferation rates and multipotent differentiation ability, MSCs are able to differentiate into cardiomyocytes [23], nerve cells [24], osteoblasts [25], hepatocytes [26], chondrocytes [27], nucleus pulposus-like cells [28], and many other cell types under suitable conditions. MSCs also play a role in immunomodulation [4, 29] by producing cytokines and cell-cell interactions, which in turn inhibit T-cell proliferation and immune responses [30] and ultimately suppress immune function [31]. Owing to these immunomodulatory functions, MSCs have potential clinical applications such as supporting hematopoiesis [32], promoting implantation of hematopoietic stem cells, treatment of patients with graft-versus-host disease [33], tissue damage repair [34] (e.g., damage to the bones, cartilage, joints [35], myocardia [36], liver, spinal cord, and nervous system), and treatment of patients with autoimmune diseases (e.g., systemic lupus erythematosus, scleroderma, and rheumatoid arthritis [37]) and as carriers for gene therapy [38]. However, MSCs of different origins may possess different biological characteristics [39–41]. In this study, we examined the characteristics and gene expression profiles of BMMSCs and SMSCs. The results showed that the MSCs from the two sources exhibited major differences in growth characteristics, cytokine secretion, and gene expression profiles, which provide insights into their different biological functions.

We found that BMMSCs and SMSCs had similar cell surface markers and multipotent differentiation capabilities; however, their proliferation rates differed significantly. The growth rate of primary BMMSCs was higher than that of SMSCs, possibly due to the existence of only two types of cells (i.e., MSCs and mononuclear hematopoietic cells) after the separation of BMMSCs. Because hematopoietic cells were nonadherent, only MSCs remained on the plate surface after the culture media were discarded. For SMSCs, however, the cellular component was more complex after separation. Despite separation of the epidermis and dermis, filtering, and other steps, the homogeneity of SMSCs was not guaranteed. Furthermore, skin cells, such as keratinocytes, fibroblasts, and vascular endothelial cells, are innately adherent. Hence, the proportion of SMSCs in the separated cell pool was not high, which led to their low primary amplification rate and their significantly lower growth rates in comparison with BMMSCs. Interestingly, the growth rate of SMSCs was significantly higher than that of BMMSCs after passaging, despite being grown under the same conditions. These results could be explained by the increased purity of SMSCs after passaging. Moreover, SMSCs exhibited significantly higher secretion of proliferative cytokines than BMMSCs. Proliferative cytokines secreted by MSCs promote the growth of not only neighboring cells, but also their own. Thus, the growth rate of later generations of SMSCs was significantly higher than that of BMMSCs.

In our study, the cultivation conditions of both types of MSCs were different. Because the purity and growth rates of the original SMSCs were low, we supplemented the cultures with 10% FBS (similar to the culture medium used for BMMSCs), bFGF, and B27 additive to the DMEM/F12 culture medium. bFGF is an important mitogenic factor that promotes cellular proliferative activity and thus promotes the growth of the original SMSCs. The B27 additive can inhibit the growth of fibroblasts, particularly those found in divided cell pools. Previously reported cultivation conditions for SMSCs have been variable. Some researchers have used medium containing no bFGF and B27 additives, only MSC growth medium alpha-modification (*α*-MEM) plus 10% FBS [42], or MSC growth medium (MSCGM) plus 10% FBS [43]. We also attempted to use culture medium without bFGF; all cultured SMSCs did not show any differences from those grown with bFGF in terms of their cellular morphologies, immunomodulatory responses, and differentiation capabilities, with the exception of the lower growth rate of the original pool of MSCs.

Although the purity of SMSCs was markedly increased after passaging, it was still lower than that of BMMSCs. This study showed that the third generation of BMMSCs reached a purity of more than 90%, while SMSCs reached only around 70% purity at the third generation and more than 90% purity at the fifth generation. This finding may be associated with the complex cellular components of the separated SMSCs; thus, we used the third generation of BMMSCs and fifth generation of SMSCs for subsequent studies. Overall, primary SMSCs grew slower than BMMSCs, while, after passaging, SMSCs grew faster than BMMSCs. Both cell types required a similar amount of time to reach the same level of purity.

To distinguish SMSCs from skin fibroblasts and to confirm that our cultured cells were MSCs, we used tissue culture techniques to cultivate skin fibroblasts. After passaging to the fifth generation and inducing differentiation, skin fibroblasts did not have the same characteristics as the SMSCs and did not differentiate into lipocytes, osteoblasts, and chondrocytes. Thus, the SMSCs and fibroblasts were indeed two different types of cells. In addition, we found that successful culturing of SMSCs required a minimum sample coverage area. If the area was too small, the number of MSCs after separation was insufficient for effective growth, resulting in culture failure. From our experience, the coverage area should be at least 2 cm^2^ for effective culturing of the desired type of cells.

The results of microarray analysis showed that BMMSCs and SMSCs displayed different gene expression. The DEGs were mainly related to the immune system and immune regulation, indicating that the immune and immune regulation functions of BMMSCs and SMSCs are different. Among the 82 DEGs involved in immune regulation, 71 were highly expressed in BMMSCs. This result suggests that the function of immune regulation is more active in BMMSCs than in SMSCs. This finding can provide guidance for the clinical use of different sources of MSCs.

In summary, in this study, we compared the morphological and molecular features of BMMSCs and SMSCs. Our results showed that these two types of MSCs exhibit some unique features. However, we have only compared two different sources of MSCs and studied only a few aspects of cell biology. Our study represents an initial investigation of the broad range of applications and sources of MSCs. More extensive studies are required to facilitate the wide application of MSCs.

## Figures and Tables

**Table 1:** Culture times for MSCs isolated from bone marrow and skin (mean ± SD, days).

Group	Time of primary culture	Time of passage 1
BMMSCs	16.35 ± 4.38	12.25 ± 4.49
SMSCs	28.85 ± 5.52	3.85 ± 1.09
*T* value	−7.935	8.124
*p* value	<0.001	<0.001

BMMSC: bone marrow-derived mesenchymal stem cell; SMSC: skin-derived mesenchymal stem cell.

**Table 2:** Cytokine levels in the culture medium of MSCs isolated from the bone marrow and skin (pg/mL).

Group				
Cytokine	Bone marrow (mean ± SD)	Skin (mean ± SD)	*T* value	*p* value
Cell proliferation-related cytokines				
EGF	52.16 ± 10.75	301.35 ± 28.92	−36.125	<0.001^*∗∗*^
SCF	99.42 ± 31.08	231.55 ± 41.10	−11.469	<0.001^*∗∗*^
bFGF	110.09 ± 27.48	163.30 ± 53.16	−3.976	<0.001^*∗∗*^
VEGF	22.18 ± 7.18	590.22 ± 64.68	−39.037	<0.001^*∗∗*^
M-CSF	66.99 ± 32.33	206.31 ± 42.20	−11.720	<0.001^*∗∗*^
G-CSF	60.21 ± 16.70	141.13 ± 13.15	−17.028	<0.001^*∗∗*^
GM-CSF	167.44 ± 36.38	194.73 ± 26.72	−2.703	0.01^*∗*^
LIF	41.62 ± 8.25	28.42 ± 10.74	4.358	<0.001^*∗∗*^
TGF-*β*1	83.54 ± 35.28	395.16 ± 80.27	−15.894	<0.001^*∗∗*^

Inflammatory cytokines				
IL-1	65.32 ± 21.71	36.29 ± 9.81	5.450	<0.001^*∗∗*^
IL-3	234.70 ± 34.81	67.03 ± 10.79	20.574	<0.001^*∗∗*^
IL-6	79.63 ± 12.65	61.67 ± 17.53	3.715	0.001^*∗∗*^
IL-7	90.24 ± 26.70	106.11 ± 21.93	−2.053	0.047^*∗*^
IL-8	55.22 ± 10.69	25.03 ± 4.56	11.614	<0.001^*∗∗*^
IL-11	124.19 ± 25.00	181.37 ± 31.74	−6.330	<0.001^*∗∗*^
HGF	57.43 ± 7.96	319.24 ± 41.03	−28.016	<0.001^*∗∗*^
TNF-*α*	24.26 ± 6.46	62.47 ± 12.41	−12.216	<0.001^*∗∗*^

EGF: epidermal growth factor; SCF: stem cell factor; bFGF: basic fibroblast growth factor; VEGF: vascular endothelial growth factor; M-CSF: macrophage colony-stimulating factor; G-CSF: granulocyte colony-stimulating factor; GM-CSF: granulocyte-macrophage colony-stimulating factor; LIF: leukemia inhibitory factor; TGF: transforming growth factor; IL: interleukin; HGF: hepatocyte growth factor; TNF: tumor necrosis factor. ^*∗*^ *p* < 0.05; ^*∗∗*^ *p* < 0.01.

**Table 3:** Top ten genes with significantly differential expression involved in immune system process and regulation of immune system process of bone marrow mesenchymal stem cells (BMMSCs) and skin mesenchymal stem cells (SMSCs).

GenBank accession	Gene symbol	Gene name	Fold change (BMMSCs versus SMSCs)	*p* value
NM_002122	HLA-DQA1	*Homo sapiens* major histocompatibility complex, class II, DQ alpha 1	611.5	0.042^*∗*^
NM_004001	FCGR2B	*Homo sapiens* Fc fragment of IgG, low affinity IIb	290.9	0.037^*∗*^
NM_000569	FCGR3A	*Homo sapiens* Fc fragment of IgG, low affinity IIIa	236.6	0.046^*∗*^
NM_022555	HLA-DRB3	*Homo sapiens* major histocompatibility complex, class II, DR beta 3	152.1	0.009^*∗∗*^
NM_001001547	CD36	*Homo sapiens* CD36 molecule (thrombospondin receptor)	133.9	0.034^*∗*^
NM_002125	HLA-DRB5	*Homo sapiens* major histocompatibility complex, class II, DR beta 5	132.0	0.014^*∗*^
NM_001774	CD37	*Homo sapiens* CD37 molecule	105.3	0.020^*∗*^
NM_006864	LILRB3	*Homo sapiens* leukocyte immunoglobulin-like receptor, subfamily B (with TM and ITIM domains), member 3	102.1	0.040^*∗*^
NM_000677	ADORA3	*Homo sapiens* adenosine A3 receptor	93.8	0.029^*∗*^
NM_002118	HLA-DMB	*Homo sapiens* major histocompatibility complex, class II, DM beta	93.3	0.035^*∗*^

^*∗*^ *p* < 0.05; ^*∗∗*^ *p* < 0.01.
